# The Comparative Root System Architecture of Declining and Non-Declining Trees in Two Apple Orchards in New York

**DOI:** 10.3390/plants12142644

**Published:** 2023-07-14

**Authors:** Alicia Serrano, Anna Wunsch, Jean Sabety, Janet van Zoeren, Michael Basedow, Mario Miranda Sazo, Marc Fuchs, Awais Khan

**Affiliations:** 1 Plant Pathology and Plant-Microbe Biology Section, School of Integrative Plant Science, Cornell University, Geneva, NY 14456, USAmarc.fuchs@cornell.edu (M.F.); 2 Cornell Cooperative Extension, Lake Ontario Fruit Program, Albion, NY 14411, USA; 3 Cornell Cooperative Extension, Eastern New York Commercial Horticulture Program, Plattsburgh, NY 12901, USA

**Keywords:** *Malus domestica*, rapid apple decline, rootstock, root traits, latent viruses

## Abstract

Rapid apple decline is a phenomenon characterized by a weakening of young apple trees in high density orchards, often followed by their quick collapse. The nature of this phenomenon remains unclear. In this work, we investigated the root system architecture (RSA) of declining and non-declining apple trees in two orchards in New York State. High-density orchard A consisted of 4-year-old ‘Honeycrisp’ on ‘Malling 9 Nic29’, and conventional orchard B consisted of 8-year-old ‘Fuji’ on ‘Budagovsky 9’. In both orchards, a negative correlation (−0.4–−0.6) was observed between RSA traits and decline symptoms, suggesting that declining trees have weaker root systems. Scion trunk diameter at the graft union, total root length, and the length of fine and coarse roots were significantly (*p* < 0.05) reduced in declining trees in both orchards. Additionally, internal trunk necrosis at, above, and below the graft union was observed in declining trees in orchard A but not in orchard B. Finally, latent viruses were not associated with decline, as their occurrence was documented in declining and non-declining trees in orchard A, but not in orchard B. Together, these results showed weakened root systems of declining trees, suggesting that these trees may experience deficiencies in water and nutrient uptake, although distinct RSA and trunk health traits between the two orchards were noticeable.

## 1. Introduction

Apple (*Malus domestica* Borkh.) is one of the most economically important temperate fruit crops worldwide [1]. In a commercial orchard, an apple tree typically consists of a clonally propagated scion cultivar grafted onto a rootstock. Scion cultivars and rootstock genotypes are selected to optimize tree performance, with the former selected for fruit quality, productivity, and disease resistance, and the latter selected for growth habit, nutrient and water uptake, cold and drought hardiness, precocity, and disease resistance, among other traits [2,3,4,5,6,7].

Most new apple orchards in the United States have transitioned to high-density plantings in recent decades to maximize productivity per unit area. Modern high-density apple orchards are established on dwarfing or semi-dwarfing rootstocks [4]. Several studies have investigated the compatibility between different scion–rootstock combinations [8,9,10]. However, little is known about the effects of the root system architecture (RSA) of rootstock genotypes on grafted scion cultivars [7]. Different rootstocks have generally different root systems, with root traits usually positively correlated with aboveground tree performance [11]. For example, larger root surface area is positively correlated with greater canopy branching, higher photosynthetic capacity, and more efficient water use [11,12,13].

The root development and root structure of apple trees are influenced by several factors including the genetics of the rootstock, soil fertility, orchard management, and planting density [14,15]. In recent years, a rapid decline of apple trees has been described in the USA and Canada [16,17,18,19,20,21]. This phenomenon is characterized by an unexplained decline of randomly distributed young apple trees in high-density orchards [20]. Symptoms typically consist of leaf curling and chlorosis at shoot tips followed by chlorosis or reddening of leaves throughout the tree canopy, and ultimately tree death. Additionally, necrotic tissue is observed in the heartwood, sapwood, and vascular cambium of declining trees, particularly near and below the graft union [20,22]. Other symptoms include a general reduction in tree growth and vigor, as well as evidence of hydraulic stress [20,21,22]. Rapid decline is characterized by the short period between initial symptom development and total tree collapse that can occur in just a few weeks, often between July and September in New York [20,22]. The rapid apple decline phenomenon has been observed in several scion–rootstock combinations, including ‘Ambrosia’, ‘Crimson Crisp’, ‘Fuji’, ‘Gala’, ‘Golden Delicious’, ‘Honeycrisp’, and ‘Salish’ scion cultivars on ‘Malling 9′ (M.9), M.9 Nic29, ‘Geneva 935′ (G.935), and ‘Malling 26′ (M.26) rootstocks [22,23,24].

Various factors potentially associated with the rapid decline phenomenon, as described above, have been previously studied in apple orchards [17,20,21,25,26]. The composition and abundance of bacterial and fungal communities were investigated in the soil, rhizosphere, roots, and shoots of declining trees, as were viruses and abiotic stressors. None of these factors was found to be significantly associated with decline. Nonetheless, necrotic lesions and wood decay symptoms were observed from the vascular cambium towards the heartwood around the graft union of declining apple trees [20,22]. Viruses, including hitherto undescribed viruses, have been reported in declining apple trees, but have not been conclusively associated with decline [17,20,25,26]. However, alphaproteobacteria in the class *Rickettsiales* were found to be less abundant in the roots of declining compared with the roots of non-declining apple trees [20]. The abundance of these bacteria usually declines in water-limiting soils, and therefore, may play a role in decline [20,27].

To build on earlier findings suggesting weak root systems in declining trees [20,25], we thoroughly compared the root system architecture (RSA) and trunk health of declining and non-declining trees in two commercial apple orchards in New York State. Here, we report our findings and discuss their implications for the etiology of the rapid apple decline phenomenon.

## 2. Results

Soil sample analyses indicated differences in the physical and chemical characteristics of the soils of the two orchards (Supplementary Table S1). The soil of orchard A had a sandy-loam texture and higher organic matter and nutrient content, higher electric conductivity, and a higher quality score than the soil of orchard B, which had a loam texture (Supplementary Table S1). Phosphorous, potassium, and zinc concentrations were more than two-fold higher in the soil of orchard A compared with the soil of orchard B (Supplementary Table S1).

Decline was observed in the two orchards selected for this study. Declining trees were predominantly randomly distributed throughout both orchards with limited aggregation. Decline severity ratings of 0–4 were attributed to trees in orchard A, while ratings of 0–3 were attributed to trees in orchard B. Overall, the vigor of declining trees was more reduced in orchard A than in orchard B. Visual observations indicated that declining trees in orchard B experienced a chronic decline, with no trees increasing in decline severity between July and November, while declining trees in orchard A experienced a more acute decline, with three of the 16 evaluated trees collapsing between July and November.

Because of the age and genetic differences between trees in orchard A (4-year-old ‘Honeycrisp’ on M.9 Nic29) and orchard B (8-year-old ‘Fuji’ on B.9), the RSA and root traits were analyzed separately (Table 1). In most cases, the root trait variables that showed significant differences between non-declining and declining trees had lower values in the declining trees, regardless of the orchard. In both orchards, average scion trunk diameter at the graft union and total root length were significantly lower (*p* < 0.05) in declining than in non-declining trees. Root dry weight was significantly lower in declining trees (*p* < 0.05) in orchard A, but not in orchard B (Table 1). The number of primary roots, root system width, and total surface area of roots were significantly lower (*p* < 0.05) in declining compared with non-declining trees in orchard A (Table 1). In orchard B, root system depth, number of root tips, and projected area of the whole root system were significantly lower (*p* < 0.05) in declining compared with non-declining trees (Table 1). In orchard B, the ratio of the rootstock to scion trunk diameter was significantly higher (*p* < 0.05) in declining trees.

**Figure 1:**
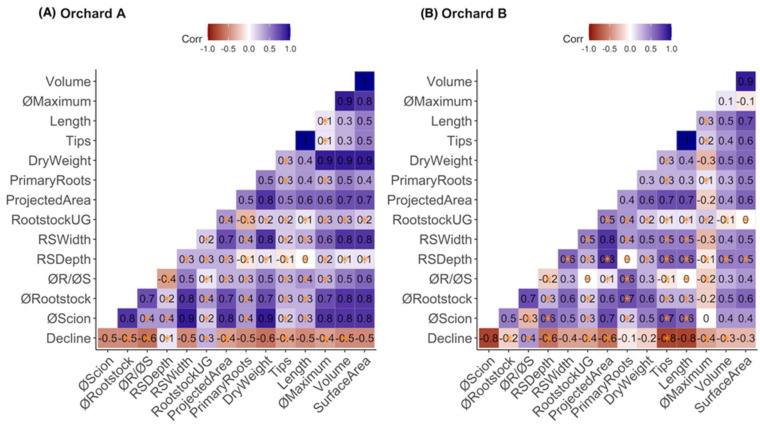
Correlation plots of root system architecture traits and tree decline in orchard A (Wayne County, NY, USA; 42°55′53″ N, 73°53′51″ W) (**A**) and orchard B (Saratoga County, NY; 43°8′40″ N, 77°13′22″ W) (**B**). The numbers in each correlation plot represent correlation values and yellow asterisks indicate significant correlation (*p* < 0.05) between RSA parameters and tree decline severity (declining trees with severity values of 2 to 4 and non-declining trees with severity values of 0 or 1). RSA parameters measured were total root volume (Volume), maximum diameter of roots (øMaximum), total root length (Length), number of root tips (Tips), dry root weight (DryWeight), number of primary roots (PrimaryRoots), projected area of the whole root system (ProjectedArea), underground rootstock stem length (RootstockUG), root system width (RSWidth), root system depth (RSDepth), ratio of rootstock trunk diameter to scion trunk diameter (øR/øS), rootstock trunk diameter at the graft union (øRootstock), scion trunk diameter at the graft union (øScion), and decline status (declining or non-declining; Decline).

Correlation analysis showed that most RSA traits were negatively correlated with decline in both orchards (Figure 1). Rootstock diameter at the graft union, projected surface area, maximum root diameter, and root volume were significantly (*p* < 0.05) negatively correlated with decline in both orchards. Root system depth and rootstock stem length were significantly (*p* < 0.05) positively correlated with decline in orchard A, but were significantly (*p* < 0.05) negatively correlated with decline in orchard B (Figure 1). The rootstock to scion trunk diameter ratio was significantly (*p* < 0.05) positively correlated with decline in orchard B, while this trait was significantly (*p* < 0.05) negatively correlated with decline in orchard A. Additionally, root system width was significantly (*p* < 0.05) negatively correlated with decline in orchard A, but not in orchard B (Figure 1).

**Figure 2:**
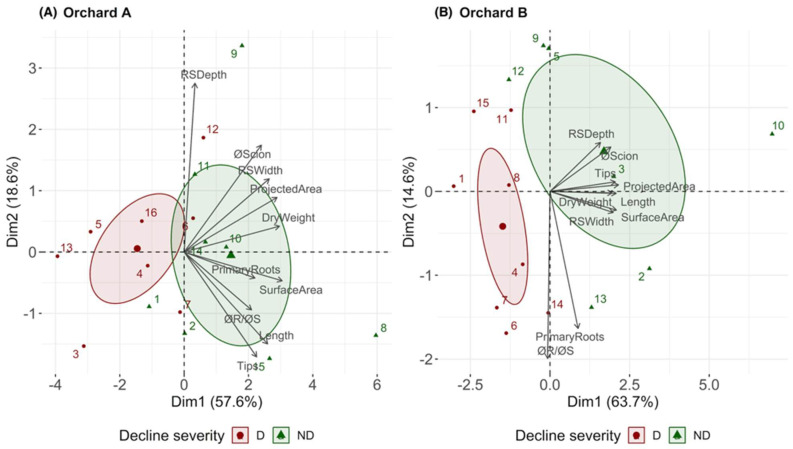
Principal component plots for orchard A (Wayne County, NY, USA; 42°55′53″ N, 73°53′51″ W) (**A**) and orchard B (Saratoga County, NY, USA; 43°8′40″ N, 77°13′22″ W) (**B**). Numbers represent individual trees. Red indicates declining trees and green indicates non-declining trees. The RSA parameters measured were scion trunk diameter at the graft union (øScion), rootstock trunk diameter at the graft union (øRootstock), ratio of rootstock trunk diameter to scion trunk diameter (øR/øS), root system depth (RSDepth), root system width (RSWidth), projected area of the whole root system (ProjectedArea), number of primary roots (PrimaryRoots), dry weight of roots (DryWeight), number of root tips (Tips), total root length (Length), and total surface area of roots (SurfaceArea).

Root traits that differed significantly (*p* < 0.05) between declining and non-declining trees were used to perform a principal component analysis (PCA). The first two PCA dimensions explained 76.2% and 78.3% of the total variability in orchards A and B, respectively (Figure 2). In both orchards, the first dimension was the most explanatory dimension for grouping trees into distinct decline categories (declining versus non-declining). This analysis demonstrated a strong correlation between decline and most root traits, as previously shown by ANOVA (Table 1) and correlation analysis (Figure 1). Dimensions 1 and 2 explained 57.6% and 18.6% of the variation in orchard A, respectively, whereas dimensions 1 and 2 explained 63.7% and 14.6% of the variation in orchard B, respectively. Focusing on dimension 1, the most explanatory dimension, scion trunk diameter at the graft union and projected area of the whole root system were the most informative root traits in both orchards (Figure 2). In contrast, root system depth was the least informative root trait in orchard A, whereas the number of primary roots and the ratio of rootstock to scion diameter were the least informative root traits in orchard B for dimension 1 (Figure 2). In both orchards, root traits of non-declining trees clustered in the positive part of the dimension 1 axis (green area), and root traits of declining trees grouped in the negative part of the dimension 1 axis (red area) (Figure 2). However, some variability was observed to a limited extent. For instance, non-declining trees 9 and 8 in orchard A differed from the rest of the non-declining trees due to their deeper root systems and higher values for either total root length, surface area, and number of root tips (non-declining tree 9), or root dry weight (non-declining tree 8) (Figure 2). Similarly, non-declining tree 10 in orchard B had higher values of RSA traits. Nonetheless, these three outlier trees remained in the positive area of the axis of dimension 1 (Figure 2).

**Figure 3:**
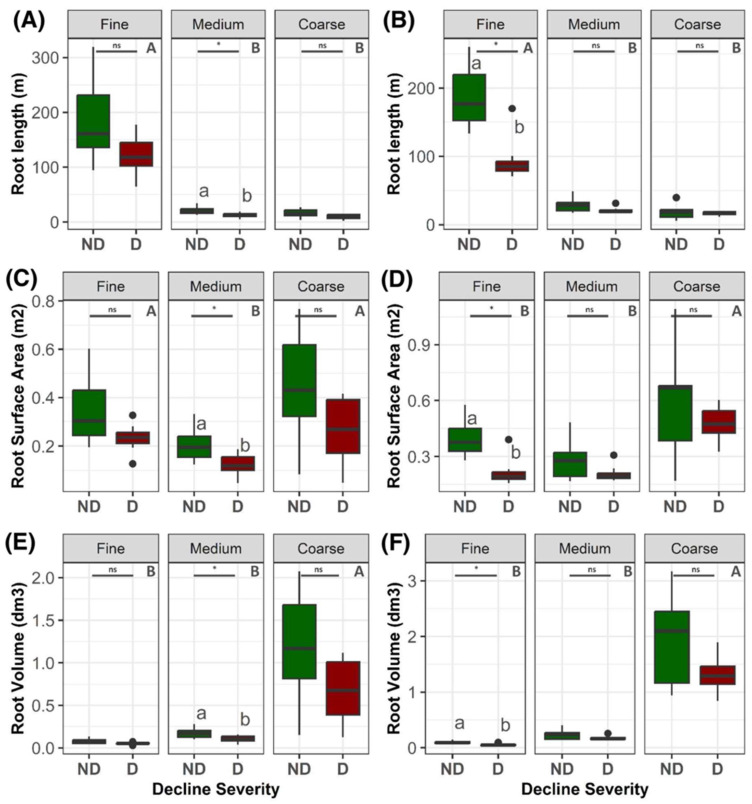
Root length, root surface area, and root volume by root size category (fine, medium, and coarse) of apple trees in orchard A (Wayne County, NY, USA; 42°55′53″ N, 73°53′51″ W) and orchard B (Saratoga County, NY, USA; 43°8′40″ N, 77°13′22″ W). Panels (**A**,**B**) represent root length, panels (**C**,**D**) represent root surface area, and panels (**E**,**F**) represent root volume of trees in orchards A and B, respectively. Fine roots (<2 mm), medium roots (2–5 mm), and coarse roots (>5 mm) were distinguished. Capital letters represent statistical differences among root sizes according to ANOVA (*p* < 0.05) and post hoc LSD tests. (*) and (ns) represent statistical differences between non-declining trees (ND, green) and declining trees (D, red) according to ANOVA (*p* < 0.05), with post hoc LSD test results represented by lowercase letters.

Further analyses revealed differences between declining and non-declining trees for total root length, surface area, and volume by orchard and root categories (Figure 3). In both orchards, most of the root length was represented by fine roots (84.3% and 75.8% in orchards A and B, respectively), which were significantly higher (*p* < 0.05) than the length of medium roots (9% and 14% in orchards A and B, respectively) and coarse roots (6.7% and 10.2% in orchards A and B, respectively). In orchard A, total root length (Figure 3A), root surface area (Figure 3C), and root volume (Figure 3E) were significantly lower (*p* < 0.05) in declining than in non-declining trees for medium roots, but not for fine and coarse roots. For instance, the mean length for medium roots was 12.28 m for declining trees and 21.25 m for non-declining trees (Figure 3A); the mean value of medium root surface area was 0.12 m^2^ for declining trees and 0.21 m^2^ for non-declining trees (Figure 3C); and the mean value of medium root volume was 0.10 dm^3^ for declining trees and 0.18 dm^3^ for non-declining trees (Figure 3E). In orchard B, significant differences between root traits of declining and non-declining trees were only detected for fine roots (*p* < 0.05). The mean value of fine root length was 94.20 m for declining trees and 191.6 m for non-declining trees (Figure 3B); the mean value of fine root surface area was 0.22 m^2^ for declining trees and 0.41 m^2^ for non-declining trees (Figure 3D); and the mean value of fine root volume was 0.06 dm^3^ for declining trees and 0.1 dm^3^ for non-declining trees (Figure 3F).

**Figure 4:**
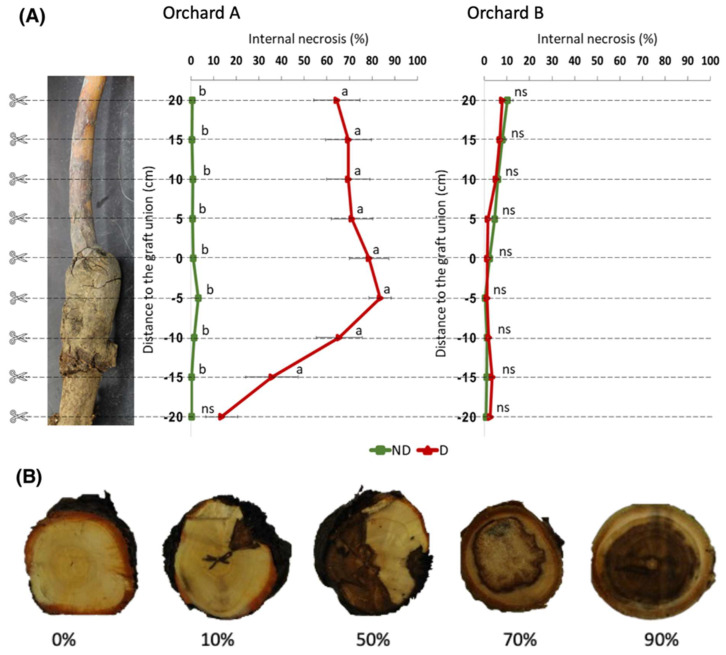
Internal trunk necrosis of declining and non-declining apple trees in orchard A (Wayne County, NY, USA; 42°55′53″ N, 73°53′51″ W) and orchard B (Saratoga County, NY, USA; 43°8′40″ N, 77°13′22″ W). (**A**) Percentage (%) area of internal trunk necrosis measured in 5 cm sections from 20 cm above to 20 cm below the graft union. Different letters (a or b) indicate statistical significance (*p* < 0.05) between non-declining (ND, green line) and declining (D, red line) trees according to ANOVA and post hoc LSD tests. Non-statistically significant differences are indicated by ns. (**B**) Representative levels of internal trunk necrosis severity in cross-sections of declining trees ranging from 90% to 0% necrotic area.

Analysis of internal trunk necrosis showed marked differences between trees in the two study orchards (Figure 4). In orchard A, declining trees showed more extensive internal necrosis than non-declining trees (Figure 4A). Necrosis was most extensive at and above the graft union with a 90% rate at 5 cm below the graft union and 63% at 20 cm above the graft union of declining trees in orchard A. Internal trunk necrosis was only 13.5% at 20 cm below the graft union. At this depth, the extent of the necrosis was not significantly different between declining and non-declining trees. In contrast, declining trees in orchard B showed minimal internal trunk necrosis, and differences between declining trees (1.21% at 5 cm below the graft union and 8.03% at 20 cm above the graft union) and non-declining trees (0.42% at 5 cm below the graft union and 10.18% at 20 cm above the graft union) were not significant (Figure 4B).

Virus testing of leaves and roots of trees revealed the presence of ACLSV in most non-declining and declining trees in orchard A (Table 2). ASGV and ASPV were each detected in a single non-declining tree, but not in declining trees, and ACLSV and ASPV co-occurred in a single non-declining tree (Table 2). None of the six viruses tested was detected in any trees in orchard B, regardless of their decline status (Table 2).

## 3. Discussion

We found significant differences between the RSA of declining and non-declining trees in two commercial apple orchards in New York State: one high-density orchard and one conventional orchard. The role of RSA traits in rapid apple decline was previously hypothesized [20,22], but empirical evidence was lacking. Our study is the first to report on substantially smaller root systems and reduced root system area in declining trees.

Some RSA traits were consistently reduced in declining trees in both orchards. For example, scion trunk diameter at the graft union and total root length were significantly reduced in declining trees compared with non-declining trees in orchards A and B (Table 1). However, most RSA traits were unique to declining trees in only one of the two orchards. Root system width, number of primary roots, dry root weight, and total surface area were significantly reduced in declining trees of orchard A, while root system depth, ratio of rootstock trunk diameter to scion trunk diameter, projected area of the whole root system, and number of root tips were significantly reduced in declining trees of orchard B (Table 1). These distinctions between declining trees in the two orchards might suggest the occurrence of different decline phenomena, among other possible factors, including the scion–rootstock combinations, tree ages, orchard systems, and soil compositions that differed between the two orchards selected for our study. All these factors can influence root systems [4,28,29], and potentially contribute to differential RSA traits between declining trees in orchards A and B. Nonetheless, despite differences in RSA traits, the root systems of declining trees in both orchards were smaller and weaker compared to those of non-declining trees.

Declining trees in both orchards had reduced fine root length and a reduced volume and surface area of coarse roots (Figure 3). Fine roots, deep roots, and the overall shape of the root system are important for water and nutrient uptake, especially in high density systems where horizontal space is limited [30,31,32]. In other crops, root system architecture and the density, diameter, branching pattern, and branching angle of various root types have been shown to be critical for plant function, optimal soil resource uptake, and interactions with biotic and abiotic factors [31,33]. Given that a robust root system influences tree vigor and architecture, yield and fruit quality, disease tolerance, and overall orchard performance and productivity, and is critical for overall plant health [11,12,13], the observed reductions in RSA traits, particularly for fine and coarse roots, may contribute to reduced water and nutrient uptake from the soil and eventually to vascular failure and tree decline.

Apple tree decline has been reported more frequently on dwarfing rootstocks, such as M.9 and B.9, which have smaller root systems than semi-dwarfing or non-dwarfing rootstocks, potentially contributing to tree weakness [20,34,35]. In addition, the high-density spacing of root systems in orchards with dwarfing rootstocks, such as in orchard A, may result in more intense competition for nutrients and water and could potentially create a more stressful environment [20]. In this study, decline was more severe in high-density orchard A than in conventional orchard B; however, as previously discussed, factors other than tree density may have contributed to differences between the two orchards.

Soil analyses of the two orchards suggested distinct texture, nutrient availability, and physicochemical properties (Supplementary Table S1). The soil of high-density orchard A had a sandy-loam texture with high nutrient content and electric conductivity, as well as a high-quality score. The soil in conventional orchard B had a loam texture, limited availability of some nutrients, and a medium quality score (Supplementary Table S1). In this orchard, unlike in orchard A, a significantly higher ratio of rootstock trunk diameter to scion trunk diameter was observed in declining trees, while no significant differences in total root surface area, number of primary roots, or root system width were observed between declining and non-declining trees (Table 1). In addition, unlike in orchard A, the root system depth was significantly lower in declining versus non-declining trees in orchard B (Table 1). These data suggest that the shorter, shallower roots of declining trees were likely less efficient at taking up the limited nutrients available in the soil of orchard B.

No significant internal trunk necrosis was observed in orchard B, but extensive necrosis was prominent between 20 cm below the graft union and 20 cm above the graft union in declining trees in orchard A. This difference could be due to distinct decline phenomena in both orchards, differences in rootstock–scion compatibility between ‘Fuji’ and B.9 compared with ‘Honeycrisp’ and M.9 Nic29, or the greater maturity of the trees in orchard B resulting in better established trees which are better able to withstand environmental stresses. The origin of the internal trunk necrosis remains unclear, although it was previously reported in declining apple trees in independent studies [17,20]. However, necrotic heartwood, sapwood, and vascular cambium suggest drastic vascular failure. Consequently, absorption and transport of water, nutrients, carbohydrates, and other assimilates would be suboptimal in such declining trees.

Internal necrosis was observed at, below, and above the graft union in declining trees in orchard A. These observations do not directly suggest a failure of the graft union, but we cannot entirely exclude this hypothesis. A failure of the graft union could impede the effective movement of water, nutrients, and carbohydrates between the grafted scion and root system of fruit trees, potentially resulting in tree decline [36]. Inadequate callus formation, elevated phenol levels, and disruption of vascular continuity, among other factors, can cause graft union failure in fruit trees [37,38,39]. The successful formation of a graft union relies on the fusion of the vascular systems of the scion and rootstock to allow water, nutrient, and macromolecule transport. However, even established graft unions can fail over time, resulting in a malformed graft union that eventually breaks down. This phenomenon is referred to as localized incompatibility. Therefore, we cannot disregard the possibility that graft union failure may be involved in the decline symptoms observed in orchard A, at least in certain trees. However, a graft union failure is not suspected in declining trees in orchard B.

Latent viruses were not associated with rapid apple decline in either of the two orchards selected for this study. Several latent viruses were found in declining but also in non-declining trees in orchard A, but none were detected in any of the trees in orchard B. Viruses were previously hypothesized to contribute to rapid apple decline [17,22,24,25,26] but experimental evidence of their direct involvement is lacking. Coinfection of ASGV and ASPV has been found to significantly impede root growth in pear cultivars [40]. It would be interesting to compare the performance of apple trees with distinct scion–rootstock combinations and single or multiple virus infections with regard to the rapid decline phenomenon.

In conclusion, this study investigated potential factors associated with the rapid apple decline phenomenon with a specific focus on apple root systems. We found that declining trees had smaller and weaker root systems compared with those of non-declining trees. Weaker root systems may have contributed to both nutrient deficiency and water stress. Internal trunk necrosis was associated with decline in only one of the two orchards selected for this study, but latent viruses were not associated with decline in either orchard. Therefore, it appears that a weakened root system is strongly associated with apple tree decline, although we cannot exclude the involvement of other factors.

## 4. Materials and Methods

### 4.1. Study Apple Orchards

**Figure 5:**
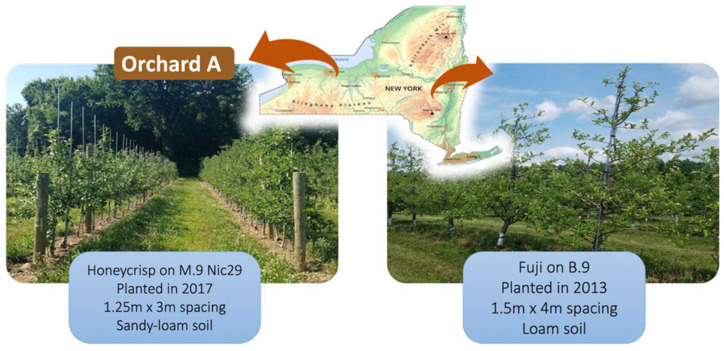
Geographic locations and layouts of orchard A (Wayne County; 42°55′53″ N, 73°53′51″ W) and orchard B (Saratoga County; 43°8′40″ N, 77°13′22″ W) selected for this study in New York, NY, USA.

Two commercial apple orchards in New York State (USA) were selected for this study. These two orchards were selected from among many other orchards experiencing diverse decline phenomena based on their distinct tree health characteristics and the growers’ willingness to support the research and have some of their trees excavated. Orchard A was planted with ‘Honeycrisp’ trees grafted onto M.9 Nic29 rootstocks in 2017 in Wayne County (42°55′53″ N, 73°53′51″ W) in western New York (Figure 5). The trees were planted at high-density spacing (1.25 m between trees and 3 m between rows) in a tall spindle production system with trellis support. Orchard B was planted with ‘Fuji’ trees grafted onto ‘Budagovsky 9’ (B.9) rootstocks in 2013 in Saratoga County (43°8′40″ N, 77°13′22″ W) in eastern New York (Figure 5). The trees in orchard B were planted at a lower density (1.5 m × 4 m) without trellis support. Conventional horticultural and pest management practices for apple orchards in New York were applied in orchards A and B throughout the duration of this work. Neither of the two orchards was irrigated.

### 4.2. Assessment of Apple Tree Decline Severity

Trees were visually evaluated from June to November 2021. Tree decline severity was visually assessed using a scale from 0 to 4, with a rating of 0 indicating no decline symptoms, and a rating of 4 corresponding to dead trees with no visible green tissue. Ratings of 1 to 3 indicated increasing decline severity, with 1 corresponding to chlorosis on a few leaves, 2 to chlorosis on most leaves, and 3 to chlorosis throughout the canopy, flagging leaves, and poor tree vigor. Based on the November assessment of decline severity, trees were grouped into two categories: a non-declining category comprised of trees with severity ratings of either 0 or 1, and a declining category comprised of trees with severity ratings of 2, 3, or 4.

### 4.3. Apple Tree Root System Sampling

A total of 31 apple trees, including 16 (eight declining and eight non-declining) in orchard A and 15 (eight declining and seven non-declining) in orchard B were excavated in November 2021. Trees were removed by hand by carefully removing the soil from around the trunks with shovels in a radius equal to half the tree spacing (60 cm in orchard A and 75 cm in orchard B) and to a depth of approximately 35 cm. After excavation, trees were cut at about 30 cm above the graft union, and the lower portion of each tree was placed in an individual bag, labelled, transported to Cornell AgriTech, and kept in cold (4 °C) storage prior to processing and evaluation of root traits.

### 4.4. Processing and Evaluation of RSA and Root Traits

**Figure 6:**
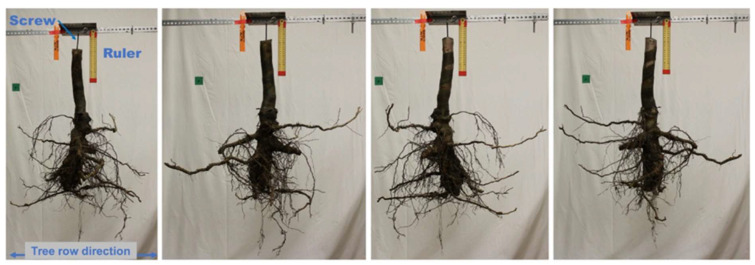
Setup used for root system imaging. Each root system was mounted on a horizontal metal rail with a screw threaded into the trunk. Four images were captured by rotating the root system from 0 to 270 degrees in four 90-degree increments. A ruler was included in each image as a size reference.

Each of the excavated root systems was washed with tap water to remove all remaining soil material. Clean root systems were mounted on a horizontal metal rail with a screw threaded into the trunk to maintain them in the same orientation as in the orchard (Figure 6). Four images of each root system were captured using a Canon EOS Rebel T5 camera (Canon, Ōta, Tokyo, Japan) by rotating the root system from 0 to 270 degrees in four 90-degree increments. A ruler was included in each image as a size reference (Figure 6). These images were analyzed using ImageJ software version 1.8 [41]. For analysis, the image scale was set using the ruler as a size reference, and each image was converted to a binary format to exclude non-root pixels from analysis. The projected area of each root system was calculated by averaging the number of root pixels over the four images taken at 0, 90, 180, and 270 degrees. The width and depth of the root systems were also measured using ImageJ. The trunk diameter of the rootstock (ØRootstock or ØR) and scion (ØScion or ØS) at the graft union were manually measured using a caliper (cm) and their ratio (ØR/ØS) was calculated by dividing the rootstock trunk diameter by the scion trunk diameter. The length of rootstock growth underground (RoostockUG) was also manually measured (cm). Next, every root branching from the rootstock stem (primary roots) was counted, cut, and scanned using an Expression 12000XL scanner (Epson Corporation, Suwa, Nagano, Japan) with the transparency unit. The scanner was set to capture 16-bit grayscale images at a 600-dpi resolution. Scanned images were analyzed using RhizoVision Explorer version 2.0.3 (https://doi.org/10.5281/zenodo.5121845 (accessed on 10 March 2023) [42]. Maximum root diameter (mm), root surface area (mm^2^), total root length (mm), and root volume (mm^3^) were measured with RhizoVision Explorer. For further analyses, roots were assigned to one of three categories based on their diameter: fine roots (<2 mm), medium roots (2–5 mm), and coarse roots (>5 mm). Total root length, surface area, and volume were assessed for each root diameter category with RhizoVision Explorer. Furthermore, the root samples were dried at 80 °C for five days and the total dry root weight was measured on a scale. Finally, the trunk was cut at 20 cm above and 20 cm below the graft union for evaluation of trunk internal necrosis. For this purpose, four cross-sections of 5 cm in thickness were taken from both the scion and rootstock segments. Each cross-section was imaged with a Canon EOS Rebel T5 camera. The internal necrosis of each cross-section was measured using ImageJ and expressed as the percentage of the necrotic wood area relative to the total area of the cross-section, as previously described [20].

### 4.5. Apple Orchard Soil Sampling

Concurrently to the tree excavations in November 2021, a soil sample was collected from each orchard using a soil sample probe (2.5 cm diameter). Each soil sample consisted of six subsamples collected near the excavated trees at a 2–15 cm depth. Physicochemical analyses of soil samples were performed at the Cornell Soil Health Lab (Ithaca, NY, USA).

### 4.6. Virus Detection

Leaf and root tissue samples of declining and non-declining trees in orchards A and B were screened using reverse transcription (RT) polymerase chain reaction (PCR) for apple chlorotic leaf spot virus (ACLSV), apple stem grooving virus (ASGV), and apple stem pitting virus (ASPV). A leaf sample consisting of 5–6 mature leaves from throughout the scaffold of each tree was collected in July 2021, and a root sample consisting of fine root tissue from throughout the excavated root system of each tree was collected in November 2021. Approximately 100 mg of tissue from each leaf and root sample was used for total RNA extraction. Total RNA was isolated using the MagMAX Viral RNA Isolation Kit (ThermoFisher Scientific, Waltham, MA, USA) with the KingFisher Flex automated extraction instrument (ThermoFisher Scientific, Waltham, MA, USA) following the manufacturers’ guidelines and stored at −80 °C prior to virus screening. Total RNA extracts were screened using multiplex RT-PCR for ACLSV, ASGV, or ASPV using primer pairs targeting a 677, 273, or 370 bp coat protein gene fragment, respectively, and a 181 bp plant mRNA (NADH dehydrogenase subunit 5) fragment as an internal control, as previously described [43]. Reactions were carried out with the OneStep Ahead RT-PCR Kit (Qiagen, Carlsbad, CA, USA) in a C1000 Touch Thermal Cycler (Bio-Rad, Hercules, CA, USA) following the manufacturers’ guidelines. Amplification products were resolved with electrophoresis on 1.5% agarose gels, stained with GelRed (Biotium, Fremont, CA, USA), and visualized under ultraviolet light. Additionally, leaf tissue samples from orchard B were screened for ACSLV, ASGV, ASPV, apple mosaic virus, tomato ringspot virus, and tobacco ringspot virus with a double-antibody sandwich enzyme-linked immunosorbent assay (DAS-ELISA) with commercial antibodies (Bioreba AG, Reinach, Switzerland).

### 4.7. Statistical Analysis

The statistical analysis of the data was performed using R (https://www.r-project.org/ (accessed on 15 August 2022). Analysis of variance (ANOVA) was applied for all RSA parameters using the aov() function of the stats R package and post hoc least significant difference (LSD) tests using the LSD.test() function from the agricolae R package [44]. The Spearman correlation between root traits and decline severity was analyzed using the cor() function from the stats R package and the relationships between these traits and the decline categories were analyzed using principal component analysis (PCA), which was performed using the factoextra R package [45].

## Figures and Tables

**Table 1:** Root system architecture (RSA) traits of declining and non-declining apple trees in orchard A (Wayne County, NY, USA; 42°55′53″ N, 73°53′51″ W) and orchard B (Saratoga County, NY, USA; 43°8′40″ N, 77°13′22″ W).

RSA Parameters ^a^	Orchard A			Orchard B		
	Non-Declining	Declining	*p*-Value	Non-Declining	Declining	*p*-Value
ØScion (mm)	28.50 ± 1.41	23.90 ± 1.14	0.025	80.50 ± 4.04	63.50 ± 2.05	0.002
Ørootstock (mm)	56.10 ± 2.25	48.80 ± 3.13	0.080	98.20 ± 7.48	91.60 ± 6.61	0.515
ØR/ØS (mm)	1.21 ± 0.05	1.03 ± 0.08	0.086	1.21 ± 0.05	1.44 ± 0.08	0.044
RSDepth (cm)	46.80 ± 2.10	47.60 ± 1.89	0.773	53.00 ± 2.08	44.40 ± 2.62	0.025
RSWidth (cm)	70.60 ± 4.24	54.90 ± 5.37	0.037	86.30 ± 7.60	69.70 ± 4.00	0.065
RootstockUG (cm)	32.50 ± 0.82	34.10 ± 0.97	0.229	32.70 ±2.01	27.70 ± 2.29	0.125
Projected Area (m^2^)	0.08 ± 0.01	0.06 ± 0.07	0.078	0.13 ± 0.02	0.08 ± 0.01	0.049
Primary Roots	30.40 ± 3.44	20.60 ± 2.62	0.041	33.70 ± 4.65	29.00 ± 2.74	0.384
Dry Weight (g)	367.00 ± 59.04	179.00 ± 28.21	0.012	805.00 ± 290.65	474.00 ± 43.13	0.249
Tips	24,021 ± 3877	17,378 ± 2100	0.154	22,716 ± 2195	11,902 ± 1347	0.001
ØMaximum (mm)	27.00 ± 2.41	24.00 ± 1.19	0.285	39.70 ± 4.80	31.40 ± 1.06	0.093
Length (m)	219.18 ± 30.79	143.06 ± 14.74	0.043	235.04 ± 25.65	131.50 ± 13.13	0.003
Surface Area (m^2^)	1.00 ± 0.14	0.61 ± 0.08	0.033	1.26 ± 0.19	0.90 ± 0.06	0.077
Volume (dm^3^)	1.43 ± 0.26	0.81 ± 0.16	0.061	2.25 ± 0.35	1.55 ± 0.12	0.064

^a^ RSA traits measured were scion trunk diameter at the graft union (øScion), rootstock trunk diameter at the graft union (øRootstock), ratio of rootstock trunk diameter to scion trunk diameter (øR/øS), root system depth (RSDepth), root system width (RSWidth), rootstock stem length underground (RootstockUG), projected area of the whole root system (Projected Area), number of primary roots (Primary Roots), dry weight of roots (Dry Weight), number of root tips (Tips), maximum root diameter (øMaximum), total root length (Length), total surface area of roots (Surface Area), and total root volume (Volume). Reported values represent mean values ± standard error. *p*-values according to the ANOVA test are indicated.

**Table 2:** Presence of latent viruses in declining and non-declining apple trees in orchard A (Wayne County, NY, USA; 42°55′53″ N, 73°53′51″ W) and orchard B (Saratoga County, NY, USA; 43°8′40″ N, 77°13′22″ W).

Orchard	Status	Total Trees	Number of Positive Samples ^a^		
			ACLSV	ASGV	ASPV
A	Non-declining	8	7	1	1
	Declining	8	7	0	0
B	Non-declining	8	0	0	0
	Declining	7	0	0	0

^a^ Number of leaf or root samples from individual apple trees that reacted positively to apple chlorotic leaf spot virus (ACLSV), apple stem grooving virus (ASGV), or apple stem pitting virus (ASPV) in RT-PCR or DAS-ELISA.

## Data Availability

There is no additional data. The data that support the results are included in the paper.

## Supplementary Materials

The following supporting information can be downloaded at: https://www.mdpi.com/article/10.3390/plants12142644/s1, Supplementary Table S1: Physicochemical properties of the soils in orchard A (Wayne County, NY, USA; 42°55′53″ N, 73°53′51″ W) and orchard B (Saratoga County, NY, USA; 43°8′40″ N, 77°13′22″ W).
