# Supplementary material for: The Comparative Root System Architecture of Declining and Non-Declining Trees in Two Apple Orchards in New York
Source: Plants (Basel). 2023 Jul 14;12(14):2644. doi: 10.3390/plants12142644 (PMC10383163; doi:10.3390/plants12142644)
Supplement: Supplementary file 1 [file plants-12-02644-s001.zip › plants-2498779-supplementary.pdf]

plants

Article

Comparative root system architecture of declining and non-declining trees in two apple orchards in New York

Supplementary Table S1. Physicochemical properties of the soils in orchard A (Wayne County, NY; 42°55'53"N, 73°53'51"W) and orchard B (Saratoga County, NY; 43°8'40"N, 77°13'22"W).

	TEXTURE			AWC	ORGANIC	pH	EC	MAJOR ELEMENTS			MINOR ELEMENTS (mg/kg)				OQS
	Sand (%)	Silt (%)	Clay (%)	g/g	MATTER (%)		(mmhos /cm)	N (%)	P (mg/kg)	K (mg/kg)	Mg	Fe	Mn	Zn	
Orchard A	54	31	14	0.19	2.9	6.2	0.13	0.15	2.8	87.6	42.1	0.5	2.4	2.2	74
	Sandy loam														*High*
Orchard B	48	34	17	0.2	2.6	6.5	0.08	0.14	0.6	32.5	62.6	0.8	1.4	0.2	46
	Loam														*Medium*

AWC: available water capacity; EC: electric conductivity; Major Elements: N (nitrogen), P (phosphorous), K (potassium); Minor Elements: Mg (magnesium), Fe (iron), Mn (manganese), Zn (zinc); and OQS: overall quality score.
